# Cisplatin ototoxicity mechanism and antagonistic intervention strategy: a scope review

**DOI:** 10.3389/fncel.2023.1197051

**Published:** 2023-06-01

**Authors:** Yingru Li, Tianyang Zhang, Qiang Song, Dekun Gao, Yue Li, Huiqun Jie, Ping Huang, Guiliang Zheng, Jun Yang, Jingchun He

**Affiliations:** ^1^Department of Otorhinolaryngology–Head and Neck Surgery, School of Medicine, Xinhua Hospital, Shanghai Jiao Tong University, Shanghai, China; ^2^School of Medicine, Ear Institute, Shanghai Jiao Tong University, Shanghai, China; ^3^Key Laboratory of Translational Medicine on Ear and Nose Diseases, Shanghai, China

**Keywords:** cisplatin, ototoxicity, apoptosis, mitophagy, antioxidants

## Abstract

Cisplatin is a first-line chemotherapeutic agent in the treatment of malignant tumors with remarkable clinical effects and low cost. However, the ototoxicity and neurotoxicity of cisplatin greatly limit its clinical application. This article reviews the possible pathways and molecular mechanisms of cisplatin trafficking from peripheral blood into the inner ear, the toxic response of cisplatin to inner ear cells, as well as the cascade reactions leading to cell death. Moreover, this article highlights the latest research progress in cisplatin resistance mechanism and cisplatin ototoxicity. Two effective protective mechanisms, anti-apoptosis and mitophagy activation, and their interaction in the inner ear are discussed. Additionally, the current clinical preventive measures and novel therapeutic agents for cisplatin ototoxicity are described. Finally, this article also forecasts the prospect of possible drug targets for mitigating cisplatin-induced ototoxicity. These include the use of antioxidants, inhibitors of transporter proteins, inhibitors of cellular pathways, combination drug delivery methods, and other mechanisms that have shown promise in preclinical studies. Further research is needed to evaluate the efficacy and safety of these approaches.

## 1. Introduction

The serendipitous discovery of cisplatin goes back to the 1960s. The physicist Barnett Rosenberg, in an investigation of the possible effects of an electric field on cell division, found that *E. coli* stopped dividing in an electrolyte with platinum electrodes, and subsequent studies revealed that this phenomenon was due to the production of a platinum compound during electrolysis, and proved that the compound also had anti-cancer activity, named it cisplatin (Guo, 2020). The main mechanism of cisplatin anti-cancer is the binding of monohydrate cisplatin complex to DNA, which interferes with DNA replication and transcription, thus inhibiting cancer cell proliferation. However, cisplatin chemotherapy can lead to various side effects such as nephrotoxicity, neurotoxicity, and ototoxicity, especially ototoxicity, which is irreversible once it occurs. The mechanism of cisplatin ototoxicity is not very clear, and therefore no well-established clinical treatment measures (Breglio et al., 2017). In view of this, this paper focuses on reviewing the latest research mechanisms and prevention strategies for cisplatin ototoxicity.

## 2. Mechanism

### 2.1. Apoptosis in cisplatin ototoxicity

For a long time, cisplatin ototoxicity has been thought to be mainly due to cisplatin-induced apoptosis of inner ear cells. Cisplatin administration reaches the stria vascularis in the lateral part of the cochlea via blood flow, and thus the marginal cells of the stria vascularis may be the earliest target of cisplatin ototoxicity (Jacobs et al., 2005). Apoptosis of marginal cells impaired stria vascularis function, resulting in loss of the endolymphatic potential and disturbance in the electrolyte composition of the endolymph environment, which are necessary for the body to maintain normal auditory function. Cisplatin crosses the blood-labyrinth barrier and enters the endolymph in the scala media via organic cation transporter 2 (OCT2) and copper transporter 1 (CTR1) in the marginal cells (Karasawa and Steyger, 2015). In the endolymph, cisplatin can enter the cochlear hair cells via various cation transport proteins and mechano-electrical transduction (MET) channels, even by passive diffusion at apical membranes, and remain for months to years. The endolymphatic potential generated by the stria vascularis can power the MET of the hair cells, making it easier for the cochlear hair cells to uptake cisplatin (Prayuenyong et al., 2021).

The main trigger of cochlear hair cell damage is the binding of the monohydrate cisplatin complex to DNA, which affects the replication and transcription of genetic material. Thus it disrupts normal intracellular metabolic activities, leading to an excessive accumulation of reactive oxygen species (ROS). Excess ROS leads to increased mitochondrial membrane permeability, impaired mitochondrial redox status, respiratory chain damage, and potassium ion efflux, ultimately leading to an irreversible cascade amplification reaction (Marullo et al., 2013). It also has been shown that in cisplatin-induced ototoxicity, resveratrol upregulates MicroRNA (miR) -455-5p to antagonize cisplatin ototoxicity by modulating the phosphatase and tensin homologue deleted on chromosome (PTEN)- Phosphatidylinositide 3-kinases (PI3K)- protein kinase B (AKT) axis, which reduces oxidative stress in hair cells, thereby protecting hearing and reducing hair cell damage (Liu et al., 2021b). Ruhl et al. (2019) found that the caspase-8 mutation promotes hair cell survival and resistance to cisplatin toxicity by the inhibition of apoptosis, demonstrating the key role of caspase-8-mediated apoptosis in the ototoxicity of cisplatin both *in vitro* and *in vivo* experimental contexts. The X-linked inhibitor of apoptosis protein (XIAP) is a key inhibitor and regulator of the last step of apoptosis signal transduction. In XIAP overexpressing mice, it was found that cisplatin-induced hair cell loss and cell structure disorder were significantly reduced (Li et al., 2022d). In addition to cisplatin-induced ototoxicity associated with apoptosis, other drug-induced hearing loss is also related to apoptosis. Apoptosis repressor with caspase recruitment domain (ARC) controls mitochondrial function and ROS levels to inhibit neomycin-induced injury of HEI-OC1 cells *in vitro* (Guan et al., 2016). Moreover, the activation of Wingless/Integrated (Wnt)/β-catenin signaling pathway inhibits caspase-mediated apoptosis and protects against neomycin-induced hair cell damage in the mouse cochlea *in vivo* (Liu et al., 2016). C-Myb is a transcription factor involved in apoptosis, and its deletion increases neomycin-induced damage to HEI-OC1 cells due to increased ROS accumulation and decreased B-cell lymphoma-2 (Bcl-2) levels (Yu et al., 2017). Activation of target of rapamycin2 (mTORC2) signaling affects PI3K/AKT related apoptosis and protects against cocaine-induced sensorineural hearing loss (Fu et al., 2022). However, inhibition of apoptosis alone cannot completely alleviate the hearing loss caused by cisplatin. More and more studies suggest that other mechanisms besides apoptosis are involved in the ototoxicity of cisplatin.

### 2.2. Autophagy in cisplatin ototoxicity

In the study of cisplatin resistance, it was found that cancer cells can reduce cisplatin therapy efficacy by triggering autophagy and autophagy-modulating agents as chemosensitizers for cisplatin therapy in cancer. Autophagy is subdivided into non-specific (bulk autophagy) and specific autophagy. The latter can remove specific cellular structures such as damaged mitochondria (mitophagy), protein aggregates, DNA, or invasive pathogens, thereby protecting cells from damage or providing the necessary energy for cellular metabolism in the case of an emergency (Gąsiorkiewicz et al., 2021). Dync1li1, a subunit of cytoplasmic dynein 1, plays a crucial role in the transportation of autophagosomes to lysosomes. Recent research has demonstrated that the knockdown of Dync1li1 can result in apoptosis-related damage to hair cells, which can ultimately lead to hearing loss (Zhang et al., 2022b). It has been demonstrated that cisplatin results in cisplatin resistance via autophagy enhancement in FaDu cells of cisplatin-resistant hypopharyngeal squamous carcinoma. Autophagy inhibitor 3-Methyladenine (3-MA) and silencing the autophagy gene Beclin-1 can block the initiation of autophagy to inhibit autophagy, thus increasing the chemosensitivity of FaDu cells to cisplatin (Zhang et al., 2021). There is a strategy that encompasses not only specific autophagy inhibition but also harnessing the process to induce autophagy-dependent cell death. Similar to cisplatin resistance, cochlear hair cell morphology was more intact and less damaged under high concentrations of cisplatin treatment compared to low concentrations of the cisplatin-treated group, which may have autophagy-related protective mechanisms involved, because of the presence of autophagosomes and higher expression of autophagic genes have been observed (Jing-chun et al., 2011). There is evidence for the regulation of autophagic mechanisms in cisplatin ototoxicity. Inhibitors of glycogen synthase kinase-3β (GSK-3), a downstream factor of AKT, can promote cochlear cell survival by selectively upregulating autophagy. Conversely, inhibitors of autophagy, such as 3-MA and chloroquine (CQ), can exacerbate the severity of cisplatin-induced hearing loss (Liu et al., 2019a; Liang et al., 2021).

Furthermore, autophagy plays a crucial role in various hair cell damage processes. The selective knockout of Foxg1 in inner ear hair cells using cre/loxp technology has been shown to affect the expression of signaling pathways, such as Wnt, Notch, insulin-like growth factors (IGF), transforming growth factor (TGF), and Hippo, all of which are relevant to the differentiation and apoptosis of hair cells (He et al., 2019). Foxg1 expression levels also have an impact on autophagy levels and the number of dead and apoptotic cells in an aging and inflammation mouse model (He et al., 2020). Low-dose aspirin has been found to activate the Forkhead Box G1 (FoxG1) autophagy pathway, promoting mitochondrial regeneration and enhancing the survival of aging inner ear hair cells (He et al., 2021). Additionally, pharmacological regulation of autophagy during aminoglycoside treatment has shown moderate alleviation of stress and hair cell loss (He et al., 2017).

### 2.3. Mitophagy in cisplatin ototoxicity

Although most previous studies have shown nuclear DNA as the target of cisplatin in cochlear cells, recent studies provide the support that mitochondria are also the major sites of cisplatin cytotoxic damage. Mitochondrial DNA (mtDNA) lacks histones compared to nuclear DNA and is more susceptible to free radicals and difficult to repair (Galluzzi et al., 2014; Sheng et al., 2019). Mitochondrial membrane potential decreases after damage, making PTEN-induced Kinase1 (PINK1) accumulate and phosphorylate ubiquitin at Ser 65 in the outer mitochondrial membrane, which then recruits and binds Parkin proteins, thereby activating E3 ligase, resulting in more ubiquitination of outer mitochondrial membrane proteins, further driving the binding of ligands such as the autophagy adaptor protein Sequestosome 1 (PSQSTM1/P62), and finally making the autophagosome fuse with the damaged mitochondria to complete the mitochondrial autophagy process (Koyano et al., 2014).

Studies have shown that mitophagy can lead to cisplatin resistance during tumor chemotherapy, thereby reducing antineoplastic efficacy. Knocking out Caveolin-1 in the A549 lung cancer cell line ultimately increases the sensitivity of lung cancer to cisplatin-induced apoptosis therapy by inhibiting Parkin-related mitophagy and amplifying cisplatin-induced mitochondria-related apoptotic signaling (Liu et al., 2020). In cisplatin-induced ototoxicity, mitochondrial autophagy likewise plays an important role. A study found that PINK1 activation and Parkin protein recruitment induced mitophagy, which antagonized cisplatin-induced apoptosis in hair cells and spiral ganglion cells (Yang et al., 2018). Marullo et al. (2013) found that cisplatin exposure induced mitophagy in auditory cells, and the knockdown of the mitophagy regulatory gene or the inhibition of mitochondrial autophagy increased the severity of cisplatin-induced cell toxicity (Cho et al., 2021). Studies have also introduced the interaction between mitophagy and apoptosis. Experiments have shown that activation of mitophagy inhibitis cisplatin-induced apoptosis in HCT116 (B) and SK-N-BE cells, and inhibition of mitochondrial autophagy aggravates cisplatin-induced apoptosis (Abdrakhmanov et al., 2019). The above results suggest that mitophagy and apoptosis may also interact in cisplatin-induced ototoxicity.

### 2.4. The interaction between apoptosis and mitophagy

Inner ear cells will inevitably develop into programmed cell death due to genetic material damage and oxidative stress induced by cisplatin. Mitochondria are the main sites where oxidative stress occurs and are an important part of the pro-apoptotic pathway. Bcl-2 family proteins and pro-apoptotic proteins such as BCL2-Associated X (Bax) can promote the release of mitochondrial cytochrome C and the formation of apoptotic bodies, thereby promoting the onset of the caspase cascades. In addition, caspases cleave autophagy-related proteins in the process of apoptosis, inhibiting the occurrence of autophagy. Interestingly, products of this cleavage often act proapoptotic in a positive feedback loop (Gąsiorkiewicz et al., 2021). However, the cell’s own protective mechanisms are also activated to inhibit the onset of the mitochondria-mediated apoptotic pathway by initiating the autophagic pathway, removing damaged mitochondria, and reducing ROS production when encountering external stimulation.

**FIGURE 1:**
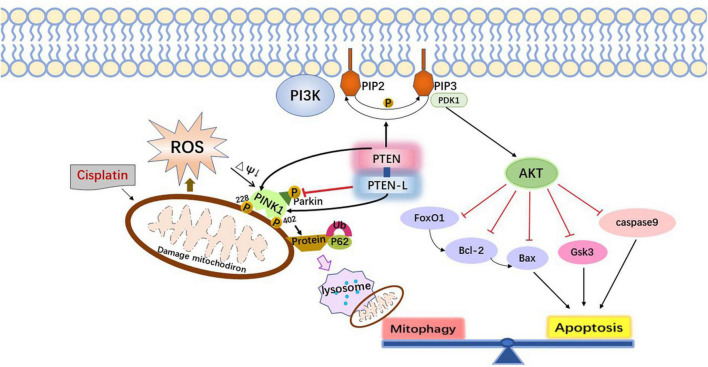
The interaction mechanism between mitophagy and apoptosis.

Therefore, the mitophagy pathway can be regulated by modulating the level of endogenous molecular substances, such as nicotinamide (NAM) and other silent information regulator T1 (SIRT1) activators, p62/SQSTM1-mediated mitophagy reducer (PMI), GSK-3β, PTEN/L, apurinic/apyrimidinic endonuclease 1. Foreign drugs such as resveratrol, fexaramine, rapamycin, and UMI-77 can also be administered to stimulate the mitophagy pathway to enhance cytoprotective effects and antagonize cisplatin ototoxicity (Georgakopoulos et al., 2017). Meanwhile, there is increasing evidence that apoptosis and mitophagy pathways overlap partly and can be co-regulated by the same biomolecules. PTEN can both activate apoptosis through the PI3K-AKT axis and regulate mitophagy through the PINK1-mediated phosphorylation of Parkin and ubiquitinated proteins (Figure 1; Wang et al., 2018). It is noteworthy that the above molecular drug targets need to comply with mild and physiological rhythms to avoid acute mitochondrial depolarization caused by cytotoxic drugs such as carbonyl cyanide 3-chlorophenylhydrazone (CCCP) and trifluoromethoxy carbonylcyanide phenylhydrazone (FCCP). Because excessive mitochondrial autophagy induced by acute mitochondrial depolarization itself can also cause programmed cell death or secondary apoptosis.

## 3. Protective measures against cisplatin ototoxicity

### 3.1. Clinical measures

There are no clinical, well-established, and physicochemical methods to effectively prevent and treat cisplatin-induced hearing damage. At present, cisplatin nephrotoxicity can be effectively prevented by hydration. But due to the complexity of the blood-labyrinth barrier function in the inner ear structure, cisplatin tends to accumulate instead of excreting. Therefore, prevention of cisplatin ototoxicity is currently more important than treatment. First of all, the indications for the use of cisplatin need to be strictly supervised, and the dose of the drug should be adjusted individually, especially the single dose and cumulative dose for pediatric patients. The method of reducing the single dose with the same total dose can make pharmacokinetics safer, lower trough concentration, and reduce the accumulation of cisplatin in the inner ear (Laurell, 2019). The combined administration of cisplatin and ototoxic drugs should be avoided to reduce the burden on the inner ear, and hearing should be closely monitored. The cisplatin dose should be reduced or even discontinued under the guidance of the clinicians once hearing impairment is detected. Thiopurine methyltransferase (TPMT), glutathione-S-transferase pi (GSTP1), cation transporters solute carrier family 22 member 2 (SLC22A2), and numerous deafness-related genes (otospiralin OTOS) have been observed to be associated with cisplatin ototoxicity in some genetics studies (Langer et al., 2020). Therefore, genetic screening can be performed in the clinical application of cisplatin to identify high-risk groups, and otoprotective agents or platinum replacement drugs can be used in advance to prevent damage in cisplatin-sensitive patients (Lee et al., 2016a). If patients have an auditory impairment, only hearing aids or cochlear implants can be applied to improve their quality of life.

Pedmark, a sodium thiosulfate (STS) formulation, is the first and only novel drug approved by the US FDA to prevent the risk of cisplatin ototoxicity in children aged 1 month and older with localized non-metastatic solid tumors. The mechanism is that STS irreversibly binds thiol covalently to free cisplatin in the blood through its sulfhydryl group, thereby eliminating cisplatin cytotoxicity. The safety and efficacy of this drug were demonstrated mainly based on two phases three clinical trials (SIOPEL6 and COG ACCLO431) (Freyer et al., 2017). The ACCLO431 clinical trial recruited participants aged 1–18 years and newly diagnosed with hepatoblastoma, germ cell tumor, medulloblastoma/central nervous system primitive neuroectodermal tumor (CNS PNET), neuroblastoma, osteosarcoma or other cancer treated with cisplatin. It demonstrated that STS significantly reduced the incidence of cisplatin-induced hearing loss (CIHL) in children and adolescents, and the effect was significant in children under 5 years of age. Although the concentration of free cisplatin in the circulation decreased significantly 4 h after administration, the SIOPEL six clinical trial found that STS administration 6 h after cisplatin chemotherapy reduced the incidence of cisplatin-induced hearing loss in children with hepatoblastoma without compromising event-free (EFS) and overall survival (OS). This suggests that delayed STS administration after cisplatin chemotherapy is effective in reducing the effect of STS on the efficacy of cisplatin chemotherapy. STS also has anti-inflammatory and antioxidant properties and can increase the levels of endogenous reducing agents such as glutathione (GSH) (Bijarnia et al., 2015; Lee et al., 2016b). In addition, local administration of STS in the inner ear increases the concentration of STS in the endolymph, which enhances the chemoprotective effect of STS against ototoxicity (Schroeder et al., 2018).

### 3.2. Research prospects

According to the pathways and mechanisms of hearing damage induced by cisplatin, numerous researchers have conducted a series of experimental studies based on different targets. Many studies have shown that these interventions are beneficial to hearing protection after cisplatin administration. Due to the unknown effect of these interventions on the anticancer activity of cisplatin and the lack of enough support for clinical trials, they have not yet been applied in the clinic. However, these findings are expected to achieve clinical translation in the future. We systematically retrieved studies published over the last year on antagonistic cisplatin ototoxicity in Pubmed database and summarized the current research hotspot and achievements. Representative studies in the past have also been described to illustrate the research basis. In general, antioxidants remain a hot area of research. And the various intracellular pathways, such as the AKT pathway, iron death pathway and mitochondria-mediated apoptosis pathway have also been extensively studied. Research on transporters has declined, likely due to poor results in associated clinical trials and the difficulty of research techniques. Other mechanisms have also emerged, such as external auditory canal cooling, changing blood labyrinth barrier permeability, novel drug delivery, and the combination of multiple mechanism drugs. The emergence of new methods will bring great vitality to the field.

#### 3.2.1. Antioxidants drugs

Cisplatin-induced auditory cell death modes including apoptosis, autophagy and ferroptosis are closely related to oxidative stress, so in preclinical studies, the antioxidant reduction pathway has been the main direction of drug development against cisplatin ototoxicity (Gentilin et al., 2019). The common antioxidants, including vitamin E, Astaxanthin (AST) (Nan et al., 2022), and alpha-lipoic Acid (Cho et al., 2022) may be clinically protective against hearing loss, but several studies have shown that antioxidants may interfere with the antineoplastic efficacy of cisplatin. However, curcumin adjuvant administration to cisplatin therapy exhibits the opposite effect on cochlear cells and cancer cells. It prefers to protect from cisplatin ototoxicity in cochlear cells by increasing the endogenous antioxidant defense system (increased nuclear factor erythroid2-related factor 2 and Heme Oxygenase-1 expression) and reducing inflammation and apoptosis (decreased NF-kB and p53) (Paciello et al., 2020). Hydrogen, a novel antioxidant substance, can exert antioxidant effects by selectively neutralizing the most toxic reactive oxygen species, hydroxyl radicals, as well as mediated by a number of fine-tuned signaling pathways, such as the nuclear factor erythroid2-related factor 2 (NRF2) pathway. Several studies have demonstrated that intraperitoneal injection of hydrogen-saturated saline, oral administration of hydrogen-saturated water, and inhalation of gaseous hydrogen can prevent hearing loss caused by cisplatin without reducing the anticancer activity confirmed by *in vitro* and *in vivo* experiments in mice (Ichihara et al., 2015; Fransson et al., 2017, 2021).

Statins are not just known to regulate blood lipid levels, but they also come into use in improving endothelial function and microcirculation, decreasing inflammation, and reducing oxidative stress under observation. A clinical trial indicated that an atorvastatin user is 53% less likely to acquire a cisplatin-induced hearing loss than a non-statin user from results analysis of 277 adults treated with cisplatin for head and neck cancer. Hence, it suggests that concurrent use of statins reduces cisplatin-induced hearing loss (Fernandez et al., 2021). Acyl-CoA synthetase long chain family member 4 (ACSL4) is the rate-limiting enzyme at the most initiation step in the arachidonic acid metabolic pathway. By using rosiglitazone to inhibit ACSL4, the production of lipid peroxide could be inhibited and the mortality of hair cells could be decreased at source satisfactorily (He et al., 2022a). Loss of Gstm1 and Gstt1 affects NRF2 expression and leads to upregulation of phase II detoxification genes. And the loss eventually changes antioxidant capacity and increases oxidative DNA and protein damage in the cochlea of cisplatin-treated Gstm1/Gstt1-DKO mice. Therefore, the presence of glutathione s-transferase mu 1 (Gstm1/Gstt1) genes is crucial for CBA/CaJ mice to resist cisplatin-induced ototoxicity (Li et al., 2022c). The overexpression of Wnt signaling in spiral ganglion neuron (SGNs) leads to increased expression of tumor protein P53-induced glycolysis and apoptosis regulator (TIGAR) and decreased levels of ROS, thereby preventing the apoptotic efflux of cascade amplification reaction (Liu et al., 2019b). Cochlear cells have an endogenous antioxidant system to combat cisplatin-induced ROS production and medical research is dedicated to helping key agents amplify the antioxidant capacity (Table 1).

**TABLE 1:** Antioxidants antagonizing cisplatin ototoxicity.

Source	References	Agent	Mechanism
Biological extract	Nan et al., 2022	Astaxanthine	Astaxanthine reduces ROS overexpression, mitochondrial dysfunction, and apoptosis via NRF2-mediated pathway.
	Paciello et al., 2020	Curcumin	Curcumin increases the endogenous antioxidant defense system (increased Nrf-2 and HO-1 expression) and reduces inflammation and apoptosis (decreased NF-kB and p53).
H_2_	Fransson et al., 2021	Inhalation of gaseous H_2_	Inhalation of 2% gaseous H_2_ have antioxidant effects, rapid distribution, and distributes systemically.
	Fransson et al., 2017	Gaseous H_2_	H_2_ inhalation reduces the OCT2 and the CTR1 intensity and synaptophysin immunoreactivity in the synapse area around the IHCs and OHCs.
Synthetic chemical	Fernandez et al., 2021	Atorvastatin	Statins come into use in improving endothelial function and microcirculation, decreasing inflammation, and reducing oxidative stress under observation.
	He et al., 2022a	ROSI (ACSL4 inhibitor)	By using ROSI to inhibit ACSL4, the production of lipid peroxide could be inhibited.
Cochlear cell components	Li et al., 2022c	GSTM1, GSTT1	Loss of Gstm1 and Gstt1 affects Nrf2 expression and leads to upregulation of phase II detoxification genes.
	Cho et al., 2022	Alpha-Lipoic Acid	Alpha-Lipoic Acid reduces the accumulation of intracellular lipid droplets to attenuates apoptosis and ferroptosis.
	Liu et al., 2019b	Wnt	The overexpression of Wnt signaling in SGNS leads to increased expression of TP53-induced glycolysis and apoptosis regulator (TIGAR) and decreased levels of ROS.

#### 3.2.2. Inhibitors of transporter proteins

Many studies have reported that cisplatin can enter cochlear hair cells via various cation transport proteins and MET machinery formed by transmembrane channel-like 1 and 2 (TMC1/2), LHFPL tetraspan subfamily member 5 protein (LHFPL5), transmembrane inner ear protein (TMIE), calcium and integrin-binding family member 2 (CIB2), and tip link proteins (Protocadherin-15 and Cadherin-23) (Li et al., 2022b). Therefore, it is possible to fundamentally eliminate the ototoxicity of cisplatin by inhibiting these channel proteins to prevent cisplatin from entering hair cells in theory.

A report suggests that the inhibition of MET by quinine or ethylene glycol tetraacetic acid (EGTA) can prevent cisplatin-induced hair cell death (Thomas et al., 2013). Recently, it is showed that genetically disrupting MET partially protects hair cells from cisplatin-induced hair cell death in mouse. And cimetidine, an inhibitor of the organic cation transporter, also partially protects hair cells from cisplatin ototoxicity, which is independent of MET (Li et al., 2022b). Another study revealed reduction of cisplatin trafficking into cochlear cells by intratympanic administration of copper sulfate, a substrate of the mammalian copper ion transport protein CTR1, 30 min before intraperitoneal administration of cisplatin may prevent cisplatin-induced hearing loss (More et al., 2010). Inhibition of OCT2 protein by cimetidine prevents cisplatin-induced nephrotoxicity and ototoxicity and they showed that OCT2 is expressed in the hair cells of the cochlea for the first time in the study (Ciarimboli et al., 2010). Lansoprazole, a proton pump inhibitor, can also inhibit OCT2-mediated transport of cisplatin. It was demonstrated that lansoprazole or loss of homolog of OCT2 alleviated the decrease of sensory hair cells in zebrafish treated with cisplatin. And using the public database containing adverse event reports to evaluate the effect of lansoprazole found that patients treated with lansoprazole have a lower incidence rate of cisplatin-induced ototoxicity compared to those without lansoprazole (Wakai et al., 2022).

However, inhibiting cation transport proteins or MET does not completely protect cochlear cells from cisplatin-induced ototoxicity, indicating that there are some other proteins working together with cisplatin uptake and ototoxicity. Cisplatin could increase Ca^2+^ influx via the transient receptor potential vanilloid receptor 1 (TRPV1), and Ca^2+^ overload subsequently activates calpain, a neutral cysteine protease, to trigger the apoptosis caspase process. It was found that ursolic acid could inhibit the TRPV1/Ca^2+^/calpain-oxidative stress pathway and alleviate oxidative stress in mice with cisplatin treatment (Di et al., 2020). At present, perhaps due to the sparse distribution in the hair cell membrane, difficult purification process and low yield, the research on transport proteins has encountered challenges. Additionally, the accuracy of research results on transport proteins is compromised when separated from the cell membrane. The complexity of interactions between multiple transport proteins makes it difficult to achieve satisfactory therapeutic effects by targeting a single type of protein. Consequently, recent research on transport proteins to disrupt cisplatin trafficking has decreased. However, with the advancement of future technologies, there is hope for new opportunities to emerge in this field (Table 2).

**TABLE 2:** Inhibitors of transporter proteins antagonizing cisplatin ototoxicity.

References	Agent	Mechanism
Thomas et al., 2013	MET	The inhibition of MET by quinine or EGTA can prevent cisplatin-induced hair cell death.
More et al., 2010	CTR1	Reduction of cisplatin trafficking into cochlear cells by intratympanic administration of copper sulfate, a substrate of the mammalian CTR1.
Ciarimboli et al., 2010	OCT2	Inhibition of OCT2 protein by cimetidine prevents cisplatin-induced ototoxicity.
Li et al., 2022b	MET or OCT	Genetically disrupting MET or OCT both partially protects hair cells from cisplatin-induced hair cell death in mouse.
Wakai et al., 2022	OCT2	Lansoprazole, a proton pump inhibitor, also can inhibit OCT2-mediated transport of CDDP.
Di et al., 2020	TRPV1	Ursolic acid inhibits the TRPV1/Ca^2+^/calpain-oxidative stress pathway.

#### 3.2.3. Inhibitors of cellular pathway

In addition, many studies have focused on apoptotic pathways, such as the Bcl-2 family, the caspase family, p53, PI3K/AKT signaling pathway and other targets. Epigallocatechin gallate (EGCG), the active component of tea polyphenols, inhibits the signal transducer and activator of transcription1 (STAT1) expression, thereby reducing UB/CO1 cell apoptosis induced by caspase-3 and Bax to protect from cisplatin-induced ototoxicity (Borse et al., 2017). Pretreatment with agmatine activates PI3K/AKT the pathway and reverses downregulated Bcl-2 (anti-apoptotic) expression and upregulated Bax (pro-apoptotic) expression in cisplatin-exposed HEI-OC1 cells and cochlear explants. And it was proved that agmatine could alleviate auditory function loss in cisplatin-exposed mice (Zhang et al., 2022a). More and more attention has been paid to the impact of epigenetics that refers to reversible and hereditary changes in gene function on auditory function loss. KDM5A regulates H3K4me3 demethylation, and its inhibitor CPI-455 regulates mitogen-activated protein kinase (MAPK) and PI3K/AKT signaling to attenuate cisplatin-induced ototoxicity (Liu et al., 2022). Rutin is a natural product from plants. It also could suppress MAPK/JNK signaling and activate PI3K/AKT signaling reducing mitochondria and auditory neural damage after cisplatin exposure (Zheng et al., 2022).

In several recent studies of cisplatin ototoxicity, investigators have focused on the more refined mitochondrial apoptosis pathway. Cisplatin trafficking into cochlear cells could overwhelm the redox balance, lead to mitochondrial outer membrane permeabilization, promote the release of mitochondrial cytochrome c mediated by Bcl-2 family, and trigger the mitochondrial apoptosis pathway mediated by apoptotic protease activating factor-1 (Apaf-1). Eupatilin effectively attenuates cisplatin-induced auditory hair cell death via the intervention of the mitochondrial apoptosis pathway *in vitro* and *in vivo* models including HEI-OC1 cells, cochlear hair cells, and zebrafish (Lu et al., 2022b). RG108 is a new non-nucleoside small molecule DNA methyl transferase (DNMT) inhibitor and could inhibit DNA methylation. It reduces cisplatin-associated loss of neuronal fibers and synapses and promotes HCs and SGNs survival by preventing mitochondrial apoptosis induced by ROS accumulation (He et al., 2022b). Puerarin, a traditional Chinese medicine extract, can also inhibit ROS overproduction, affect the Bcl-2 family and AKT level to regulate the mitochondrial apoptosis pathway and protect hair cells (Xu et al., 2022).

Autophagy is also an important cellular pathway for antagonistic strategy of cisplatin ototoxicity. Meclofenamic acid reduces ROS accumulation and apoptosis and improves hair cell-like HEI-OC1 cell viability by inhibiting cisplatin-induced excessive autophagy (Li et al., 2018). And trehalose prevents cisplatin-induced cochlear hair cell damage by autophagy, which is attributed to increasing nuclear translocation of transcription factor EB (TFEB) (Li et al., 2022e). Epigenetics is of high interest and can affect cisplatin-induced ototoxicity by acting on different signaling pathways. YTH N6-methyladenosine RNA binding protein F1 (YTHDF1) can protect hair cells from cisplatin-induced ototoxicity by regulating RNA metabolism by binding to a specific m6A motif and promoting autophagy by increasing autophagy protein 14 (AG14) translation in HEI-OC1 cells (Huang et al., 2022). Because apoptosis and autophagy interact in auditory hair cells after cisplatin administration, U0126, a specific inhibitor of the extracellular signal-regulated protein kinases 1 and 2 (ERK1/2) signaling pathway, has neuroprotective effects, and its pre-treatment inhibits cisplatin-induced apoptosis and autophagy in HEI-OC1 cells and cochlear hair cells (Wang et al., 2021). Modulating the activation of the antioxidant enzyme peroxiredoxin 1 (PRDX1), either through upregulation or downregulation, can have an impact on both ROS accumulation and autophagy, ultimately affecting the viability of SGNs following cisplatin treatment (Liu et al., 2021a).

In addition, the NRF2 signaling pathway is also closely related to cisplatin-induced cochlear cell death. NRF2 knockout inhibited ferroptosis and prevented cisplatin-induced hearing loss by increasing glutathione peroxidase 4 (GPX4) protein levels and decreasing transferrin receptor 1 (TfR1) protein levels (Wang et al., 2022). Polydatin (3,4′,5-trihydroxystilbene-3-β-d-glucoside) could attenuate cisplatin-induced hearing loss in guinea pigs. The mechanism is that polydatin can ameliorate oxidative stress and apoptosis injury by activating the NRF2/HO-1 signaling pathway (Li et al., 2022a). Just a few months ago, it was indicated that estradiol also activated the NRF2 signaling pathway and the transcription of genes encoding phase II detoxification enzymes, thereby ameliorating cisplatin-induced hair cell loss in cochlear explants of C57BL/6 mice (Adachi et al., 2023).

Overall, multiple cellular pathways are involved in resistance to cisplatin injury in cochlear cells. 5,7-Dihydroxy-4-methylcoumarin (D4M) markedly regulated p-c-Jun N-terminal kinase (p-JNK) and elevated the expression ratio of p-FoxO1/FoxO1, thus attenuating SGNs injury, mitochondrial dysfunction, ROS accumulation and cisplatin-induced caspase-dependent apoptosis (Li et al., 2023). As a calcium channel antagonist with lipophilic properties, nimodipine pre-treatment could significantly reduce cisplatin-induced apoptosis via regulating LIM domain only 4 (LMO4) levels and activating associated Akt, cAMP-response element binding protein (CREB), and Signal transducer and activator of transcription 3 (Stat3) protein levels (Fritzsche et al., 2022; Table 3).

**TABLE 3:** Inhibitors of cellular pathway antagonizing cisplatin ototoxicity.

Cellular pathway	References	Agent	Mechanism
Apoptosis	Borse et al., 2017	Epigallocatechin-3-gallate	Epigallocatechin-3-gallate inhibits the signal transducer and activator of transcription1 (STAT1) expression, thereby reducing UB/CO1 cell apoptosis induced by caspase-3 and Bax.
	Zhang et al., 2022a	Agmatine	Agmatine upregulate PI3K/AKT pathway to alleviate cisplatin-induced cochleae cell apoptosis.
	Liu et al., 2022	CPI-455	KDM5A regulates H3K4me3 demethylation, and its inhibitor CPI-455 regulate MAPK and PI3K/AKT signaling to attenuate cisplatin-induced ototoxicity.
	Zheng et al., 2022	Rutin	Rutin could suppress MAPK/JNK signaling and activate PI3K/AKT signaling reducing mitochondria and auditory neural damage after cisplatin exposure.
Mitochondrial apoptosis.	Lu et al., 2022b	Eupatilin	Eupatilin intervenes mitochondrial apoptosis pathway *in vitro* and *in vivo* models.
	He et al., 2022b	RG108	RG108 protects mitochondrial function through preventing ROS accumulation induced mitochondrial apoptosis.
	Xu et al., 2022	Puerarin	Puerarin, a traditional Chinese medicine extract, also can inhibit ROS overproduction, regulate Bcl-2 family and AKT level to regulate mitochondrial apoptosis pathway.
Autophagy	Li et al., 2018	Meclofenamic Acid	Meclofenamic acid reduces ROS accumulation and apoptosis and improves hair cell-like HEI-OC1 cell viability by inhibiting cisplatin-induced excessive autophagy.
	Li et al., 2022e	Trehalose	Trehalose protects against cisplatin-induced cochlear hair cell damage by autophagy attributed to increasing nuclear translocation of transcription factor EB (TFEB).
	Huang et al., 2022	YTHDF1	YTHDF1 can regulate RNA metabolism through binding to specific m6A motifs and promote autophagy via increased AG14 translation among HEI-OC1 cells.
	Liu et al., 2021a	PRDX1	Modulating the activation of the antioxidant enzyme peroxiredoxin 1 (PRDX1) can have an impact on both ROS accumulation and autophagy.
Apoptosis and autophagy	Wang et al., 2021	U0126	U0126, a specific inhibitor of the extracellular signal-regulated protein kinases 1 and 2 (ERK1/2) signaling pathway, has neuroprotective effects.
Nrf2 signaling pathway	Wang et al., 2022	Nrf2	Nrf2 knockout inhibits ferroptosis through increasing GPX4 protein levels and decreasing TfR1 protein levels.
	Li et al., 2022a	Polydatin	Polydatin can ameliorate oxidative stress and apoptosis injury through activating the Nrf2/HO-1 signaling pathway.
	Adachi et al., 2023	Estradiol	Estradiol activated the Nrf2 signaling pathway and the transcription of genes encoding phase II detoxification enzymes.
JNK/FoxO1 signaling pathway	Li et al., 2023	D4M	5,7-Dihydroxy-4-methylcoumarin (D4M)markedly regulated p-JNK and elevated the expression ratio of p-FoxO1/FoxO1.
LMO4 signaling pathway	Fritzsche et al., 2022	Nimodipine	Nimodipine pre-treatment regulates LMO4 levels and activates of associated Akt, CREB and Stat3 protein levels.

#### 3.2.4. Other novel mechanisms

Due to the lack of significant effect of individual drugs in preventing from cisplatin-induced ototoxicity, researchers have been exploring combination drug delivery methods over the past year. The method can combine the advantages of different drugs and use materials as carriers or packaging to give the combination more suitable pharmacokinetic properties. Alpha-lipoic acid, phosphatidylcholine and a gelatin-curcumin conjugate via direct sonication become hybrid liposomes (LA@PCGC) with a spherical shape and a mean diameter of 25 nm. LA@PCGC was injected into company with alginate-based hydrogel carrier to guarantee an appropriate residence time for LA within the administration site. These properties make it become a promising tool for protection from cisplatin-induced ototoxicity (Curcio et al., 2022). The combination of Pifithrin-alpha (PFT-α), d-met, and Neurotrophin-3 (NT3) consisting of an antioxidative, neurotrophic and anti-apoptotic factor given prior to cisplatin exposure has a significant cellular protective effect on cisplatin-induced death (Febles et al., 2022). As an improved drug delivery system, astaxanthin (ATX) encapsulated in ROS-responsive nanoparticles (PPS-NT)protect HCs and SGN from cisplatin-induced ototoxicity. Compared with drug administration alone, ATX-PPS-NP combined the effect of ROS consumption with the antioxidant effect and verified a quick penetration across the round window membrane in guinea pigs (Gu et al., 2022). Another liquid crystalline nanoparticles combined with dexamethasone also have a positive effect on prevention of cisplatin ototoxicity which may be due to increasing drug diffusion and improvement of defects in solubility and bioavailability of dexamethasone (Valente et al., 2022). Adeno-associated virus (AAV) vector-mediated gene therapy has been approved in the kinds of organs with high safety and efficiency. AAV-inner ear (AAV-ie) and mutant AAV-ie-K558R were designed to deliver gene in the cochlea cells for treating inherited diseases and have proved that they can transduce hair cells and supporting cells in the cochleae of neonatal mice (Tan et al., 2019; Tao et al., 2022).

Just like the blood-brain barrier (BBB), blood-labyrinthic barrier (BLB) can effectively isolate the blood from the inner ear labyrinth, preventing pathogens or toxic microorganisms from entering inner ear cells directly from the blood. It is one of the main pathways for blood-borne substances to enter the inner ear. The integrity and permeability of the BLB are important factors in maintaining cochlear homeostasis. Cisplatin could induce the damage of bovine cochlear pericytes (BCPs) that regulate BLB permeability, thereby disrupting cochlear homeostasis. Additional platelet-derived growth factor (PDGF-BB) induced recovery of BCP proliferation in the presence of cisplatin (Anfuso et al., 2022). Mannitol intravenous injections at t = 6 h reduced cisplatin ototoxicity by increasing the permeability of the blood labyrinth barrier (BLB) without exacerbating cisplatin trafficking into cochlear cells at t = 0 h in rats. The ability of mannitol as a new drug delivery technology combined with ear protection drugs may become a viable strategy (Noman et al., 2022).

There are always new mechanisms and approaches being developed in the field of the prevention of cisplatin ototoxicity, and researchers are constantly exploring new ways to improve the efficiency and effectiveness of drugs to antagonize cisplatin-induced ototoxicity. As such, there are many innovative methods and mechanisms waiting to be tested and implemented, offering exciting new possibilities for the future. It has also been demonstrated that cooling the external auditory canal with water or ear bars significantly reduces the auditory brainstem response (ABR) and distortion product otoacoustic emissions (DPOAE) threshold shift under cisplatin exposure. This phenomenon may be related to the protective mechanisms of neurological injury mediated by hypothermia or local cooling. Underlying mechanisms include blockage of upstream and downstream cell death pathways by reducing the uptake of cisplatin in cochlear cells, production of reactive oxygen species, and inflammation factor (Stanford et al., 2021). It was also discovered that aspirin administration may reduce cochlear metabolism and lead to reversible temporary threshold shift (TTS), thereby improving resilience of the cochlea to ototoxicity of cisplatin (Tzelnick et al., 2022). Endoplasmic reticulum (ER) stress also triggers cisplatin-induced apoptosis following activation of caspase 12 localized at the cytosolic membrane of ER. Both activating eukaryotic translation initiation factor2α (eIF2α) signaling pathway and its dephosphorylation inhibitor salubrinal significantly reduced cisplatin-induced cochlear hair cell ERS levels and attenuated cell injury, while eIF2α knockdown inhibited the protective effect (Lu et al., 2022a). A novel acetophenone compound can act as nucleophilic substitutes for cisplatin in platinum-cysteine thiolate site interactions which is responsible for cisplatin ototoxicity. And acetophenones have a longer half-life period and can easily enter the cell, making them readily bioavailable against end-organ damage of cisplatin (Geohagen et al., 2023). Heat shock pretreatment could lead to the overexpression of 70 kilodalton heat shock proteins (HSP70) in exosomes derived from bone marrow mesenchymal stem cells from heat shock precondition, which could alleviate cisplatin-induced hair cell loss mediated by NOD-like receptor thermal protein domain associated protein 3 (NLRP3) inflammasome (Yang et al., 2022). Extra cellular vesicles are composed of exosomes, microvesicles, apoptotic bodies, and oncosomes. The Morpho butterfly wing-integrated micro vortex biochip has the ability to isolate extracellular vesicles through the orderly arrangement of periodic nanostructures on Menelaus wings and the lipid bilayer membrane structure of the nanoprobe (Han et al., 2020).

In addition to enhancing the detoxification function of hair cells to alleviate cisplatin-induced hair cell death, promoting the trans-differentiation of supporting cells into new hair cells is a promising and effective strategy for alleviating cisplatin-induced hearing loss. Downregulation of the cell cycle pathway and the Notch signaling pathway, likely induced by Foxg1 knockdown in supporting cells, has been shown to lead to the generation of new hair cells (Zhang et al., 2020). Furthermore, AAV-ie-mediated overexpression of the guanine nucleotide exchange factor (Net1) in supporting cells has been demonstrated to induce supporting cell proliferation and hair cell regeneration, probably rescuing hair cell damage (Zhang et al., 2023; Table 4).

**TABLE 4:** Other novel mechanisms antagonizing cisplatin ototoxicity.

Classification	References	Agent	Mechanism
Combined drug administration	Curcio et al., 2022	LA@PCGC	LA@PCGC was injected in company with alginate-based hydrogel carrier guaranteeing an appropriate residence time for LA within the administration site.
	Febles et al., 2022	Combination of PFT-α, d-met, and NT3	The combination consisted of an antioxidant, neurotrophin and anti-apoptotic given prior to cisplatin exposure have significant cellular protective effect on cisplatin-induced death.
	Gu et al., 2022	ATX-PPS-NP	ATX-PPS-NP combines the effect of ROS consuming with the antioxidant effect and verifies a quick penetration across the round window membrane in guinea pigs.
	Valente et al., 2022	The conjugation of dexamethasone with nanoparticles	Another liquid crystalline nanoparticles combined with dexamethasone increases the drug diffusion and improves solubility and bioavailability of dexamethasone.
Regulating BLB permeability	Anfuso et al., 2022	Pericytes of Stria Vascularis	Cisplatin could induce the damage of bovine cochlear pericytes (BCPs) that regulate BLB permeability. Additional platelet-derived growth factor (PDGF-BB) induced a recovery of BCPs proliferation.
	Noman et al., 2022	Mannitol	Mannitol intravenous injections at t = 6 h reduced cisplatin ototoxicity through increasing the permeability of the blood labyrinth barrier (BLB).
Reducing cochlear metabolism	Stanford et al., 2021	Cool	Underlying mechanisms include blockage of upstream and downstream cell death pathways by reducing the uptake of cisplatin in cochlear cells, production of reactive oxygen species, and inflammation factor
	Tzelnick et al., 2022	Aspirin	Aspirin administration may could reduce cochlear metabolism and lead to reversible temporary threshold shift (TTS).
Affecting diverse cellular process	Lu et al., 2022a	Salubrinal	Both activating eukaryotic translation initiation factor2α (eIF2α) signaling pathway and its dephosphorylation inhibitor salubrinal significantly reduced endoplasmic reticulum and attenuated cell injury.
	Geohagen et al., 2023	Acetophenone	A novel acetophenone compounds can act as nucleophilic substitutes for cisplatin in platinum-cysteine thiolate site interactions which is responsible for cisplatin ototoxicity. And acetophenones have longer half-life period and can easily enter the cell.
	Yang et al., 2022	HSP70-BMSC-Exosomes	Overexpression of 70 kilodalton heat shock proteins (HSP70) in exosomes could alleviate cisplatin-induced hair cell loss mediated by NLRP3 inflammasome.
Trans-differentiation	Zhang et al., 2020	Foxg1	Foxg1 knockdown in supporting cells has been shown to lead to the generation of new hair cells.
	Zhang et al., 2023	Net1	Overexpression of the Guanine nucleotide exchange factor (Net1) induce supporting cell proliferation and hair cell regeneration.

## 4. Conclusion

The development of drugs to antagonize cisplatin-induced ototoxicity will play a crucial role in advanced cancer chemotherapy. Although Pedmark has been successfully approved for sale as a symptomatic drug, further confirmation of its clinical effectiveness is needed after its release in the clinical application. Cisplatin activates multiple signaling pathways in inner ear cells, forming a complex interactive network. Therefore, the development of drugs that target common points of these crosstalk pathways is a novel approach to antagonize cisplatin-induced ototoxicity without affecting antineoplastic efficacy.

## Author contributions

YiL: conceptualization, data curation, formal analysis, funding acquisition, investigation, methodology, project administration, resources, software, supervision, validation, visualization, and writing—original draft. TZ: formal analysis, investigation, methodology, project administration, resources, software, supervision, validation, visualization, and writing—original draft. QS: formal analysis, funding acquisition, investigation, methodology, project administration, resources, and supervision. DG: formal analysis, funding acquisition, and investigation. YuL: visualization and data curation. HJ and PH: data curation, formal analysis, and funding acquisition. GZ: funding acquisition, investigation, and methodology. JY: conceptualization and formal analysis. JH: conceptualization, formal analysis, funding acquisition, investigation, methodology, project administration, resources, validation, and writing—review and editing. All authors contributed to the article and approved the submitted version.
